# Evolution of Plant Phenotypes, from Genomes to Traits

**DOI:** 10.1534/g3.115.025502

**Published:** 2016-02-11

**Authors:** Josep M. Casacuberta, Scott Jackson, Olivier Panaud, Michael Purugganan, Jonathan Wendel

**Affiliations:** * Plant and Animals Genomics Program, Center for Research in Agricultural Genomics, CRAG (Consejo Superior de Investigaciones Científicas, Institut de Recerca i Tecnologia Agrícola, Universitat Autònoma de Barcelona, Universitat de Barcelona), 08193 Barcelona, Spain; † Center for Applied Genetic Technologies University of Georgia, Athens, Georgia 30602; ‡ Laboratoire Génome et Développement des Plantes Université de Perpignan Via Domitia, 66860 Perpignan, France; § Center for Genomics and Systems Biology New York University, New York 10003; ** Department of Ecology, Evolution, and Organismal Biology, Iowa State University, Ames, Iowa 50011

Connecting genotype to phenotype is a grand challenge of biology. Over the past 50 years, there have been numerous and powerful advances to meet this challenge, including next-generation sequencing approaches (Jackson *et al.* 2011), molecular genetic mapping techniques, computational modeling, and the integration of evolutionary theory and tools. In plants, the long history of domestication and breeding has provided multiple insights into the genotype–phenotype equation (Meyer and Purugganan 2013; Olsen and Wendel 2013). Domestication and breeding provide unique systems with which to study the evolution of traits and adaptation to new environments. At present, agriculture faces unprecedented challenges, with the need to continue to increase food quality and food production for a population that will likely exceed 9 billion by 2050, combined with the urgent need to make agriculture more sustainable in an environment that will be altered by climate change (Diouf 2009). Crop wild relatives, however, have evolved under ecological settings that often are more extreme than those under cultivation and thus represent a reservoir of useful adaptive traits. This genetic diversity has mostly been untapped because of a lack of appropriate tools, both at the genetic level and in describing plant phenotypes and adaptation (Mace *et al.* 2013). In this context, crop improvement needs to undergo a qualitative leap forward by exploiting the knowledge from the interface of the fields of molecular evolution, bioinformatics, plant physiology, and genetics.

With the objective of reviewing the most recent advances and identifying unanswered questions at this interface, a group of scientists met in Barcelona in March 2015 for a workshop organized by B-Debate (www.bdebate.org) and the Center for Research in Agricultural Genomics (CRAG, www.cragenomica.es), with the support of the US National Science Foundation. The meeting was divided into three scientific sessions. The first concentrated on the mechanisms that generate genomic diversity in plants, with a particular emphasis on transposable elements and polyploidy, while the second and third sessions were devoted to the evolution of plant phenotypes in wild and domesticated species, and to domestication and plant improvement processes, respectively.

## Mechanisms Generating Genome Variability: Polyploidy and Transposable Elements

The sequencing of complete plant genomes has allowed new insights into plant genome architecture and evolution. Two of the most important findings are that the evolutionary histories of plants include multiple rounds of whole genome doubling (WGD) (Wendel 2015), and that the major contributor to plant genome dynamics and genome size evolution and differentiation has been the differential and lineage-specific proliferation (and deletion) of various transposable elements (TEs) (El Baidouri and Panaud 2013; Lisch 2013; Sanseverino *et al.* 2015). These processes are now being shown to be associated with phenotypic diversification in plants, and thus represent an important link between genotype and phenotype. Several speakers presented new evidence for the important role these two mechanisms play in genome plasticity. Pamela Soltis (University of Florida) explored phenotypic variation and, in particular, ecological niche models, for allotetraploid species of *Tragopogon* and their diploid parents. She showed that following polyploidization a fraction of the DNA is lost, but there is great variability with respect to the actual loci lost among individuals. Allopolyploid *Tragopogon* individuals are mosaics of parental, intermediate, and novel phenotypes, and *Tragopogon* populations are therefore genetically and phenotypically variable, exhibiting novelty for a range of features, as observed in other polyploids as well (Soltis *et al.* 2014, 2015). Polyloidy presents challenges for chromosome pairing because of the need for proper chromosome segregation during meiosis, and neopolyploids often show defects in meiotic chromosome segregation and low fertility. One possible evolutionary trajectory for meiotic stability is to reduce the number of crossovers per chromosome, as Kirsten Bomblies (Harvard University, now at the John Innes Center, UK) suggested. She presented data on *Arabidopsis arenosa*, a species with diploid and polyploid populations, showing that in polyploid populations, different genes involved in meiosis and the regulation of crossover formation show signs of selection. Doug Soltis (University of Florida) emphasized the importance of WGD events in angiosperm evolution. He tested whether WGDs coincide with radiation events, and he presented data to support a lag-time between WGD events and increases in species diversification (Tank *et al.* 2015; Soltis *et al.* 2015). Moreover, several major ancient WGDs in angiosperms seem to be associated with morphological or chemical novelties that may be recognized as key innovations. Malika Ainouche (Université de Rennes, France) presented data on polyploidy in the grass genus *Spartina*, which has undergone repeated cycles of interspecific hybridization and whole-genome duplication (Ainouche *et al.* 2012), resulting in the formation of the invasive allododecaploid *Spartina anglica* that originated in Western Europe during the 19th century. A remarkable aspect of this system is that genome merger and doubling have led to novel and important ecological functions, and the evolution of new, heritable biochemical abilities in some polyploid lineages colonizing low-marsh areas.

The second important mechanism regarding the origin of genome variability reported at the meeting was the mobilization of TEs. Michele Morgante (Università di Udine, Italy) introduced the idea that the genome of a plant species can be divided into the pan genome (PG), which includes the core genomic features common to all individuals of a species, and the dispensable genome (DG), composed of genetic elements that are not shared by all individuals. The DG comprises the youngest and most dynamic fraction of the genome, and may be a key factor for adaptation. TEs are an important part of the DG, and Morgante showed that they can contribute not only to structural but also epigenetic differences between alleles in crops such as grapevine. Damon Lisch (Purdue University), presented data on the developmental control of TE silencing. He showed that during the transition from juvenile to adult stages of maize leaves, the silencing of some transposons, such as *MuDR*, is partially released, as are genes regulated by *trans*-acting RNA silencing mechanisms. His data stressed the close intertwining of TE and gene silencing, and the high sensitivity of these processes to developmental changes (Li *et al.* 2010). Tetsuji Kakutani (National Institute of Genetics, Japan) presented new data on TE silencing in *Arabidopsis* and, in particular, on the mechanisms that may allow TEs to escape this control. He showed that similar to the situation in some viruses, TEs can encode suppressors of silencing. His data suggested that different families of TEs would encode different suppressors that would be effective only on elements of the same family (Ito and Kakutani 2014). Finally, Marie Mirouze (Institut de Recherche pour le Développement, France) presented a novel approach to study TE dynamics based on the study of circularized elements, which are by-products of the transposition process. She proposed that this sensitive and unbiased method could be used to characterize the mobile component of genome, the mobilome, of a particular tissue or stress condition. This session ended with a general discussion about the possibility of using this method to compare the TE content of related diploid and polyploid species. Also discussed was the impact of TEs on chromosome pairing in polyploids and on differential loss or retention of duplicated gene copies following WGD events.

## Evolution of Plant Phenotypes in Wild and Domesticated Species

The second session of the meeting was devoted to the analysis of the genetic basis of the evolution of important traits in both wild and crop species. Olivier Loudet (Institut National de la Recherche Agronomique, Institut Jean-Pierre Bourgin, France) presented the work that his lab is doing to decipher the complexity of the quantitative natural variation in response to environmental changes. He is using a QTL mapping approach with crosses between individuals from wild populations. He discussed the difficulties of analyzing quantitative variation of small effects with enough precision to tackle the genotype by environmental interactions, and presented his laboratory phenotyping facilities that have helped them to start dissecting the quantitative genetics of plant growth (Tisné *et al.* 2013). Ortrun Mittelsten-Scheid (Gregor Mendel Institute, Vienna, Austria) presented her recent work on the characterization of epialleles, their stability, and their interaction in polyploids using *Arabidopsis* as a model. She described a silencing event of a transgene that depends on the presence of repeats and that only occurs in tetraploids. She concluded that any structural change in the genome may have epigenetic consequences and could create diversity, and stressed the tight link between genetic and epigenetic changes (Foerster *et al.* 2011). She also showed that, although epialleles are reversible, they can also be stable for quite long periods of time.

Mapping and isolating genes that underlie complex traits continues to be a major area of study in plant biology. Carlos Alonso-Blanco (Centro Nacional Biotechnología, Consejo Superior de Investigaciones Científicas, Spain) presented work on the molecular basis of quantitative traits, using *Arabidopsis* as a model. He reported on the characterization of loci responsible for quantitative variation of flowering time, temperature responses of vegetative growth, and arsenate tolerance, and he discussed the importance of the genetic and environmental interactions for generating quantitative variation in changing environments (Alonso-Blanco and Méndez-Vigo 2014; Zhu *et al.* 2015). John Willis (Duke University) presented the work of his laboratory on the adaptation of *Mimulus guttatus* to serpentine and copper mine tailing soils. In particular, his group is interested in determining whether the same or different genes have been used in the repeated adaptation of this species to these extreme soils. Christina Richards (University of South Florida) presented her work on the analysis of the role of genetic and epigenetic variation in ecological adaptation, using different species such as *Spartina* and *Fallopia*, and discussed to what extent epigenetic adaptation was similar to genetic selection (Richards *et al.* 2012). Amy Lawton-Rauh (Clemson University) discussed a novel weedy rice origin in a region where weedy rice was previously eradicated and reemerged where rice is cultivated, despite a lack of endemic *Oryza* species and strict management practices. She proposed that this weedy rice has not been reintroduced but, instead, originated by dedomestication of cultivated rice in a process called endoferality. She discussed the traits and underlying genes that are modified during dedomestication. Finally, Regina Baucom (University of Michigan) gave a talk on the costs of maintaining an ecologically relevant trait in nature. She shared her recent analysis on adaptation of weedy plants in agroecosystems to develop herbicide resistance, and discussed the nature of the costs that accompany a new adaptive trait (Debban *et al.* 2015).

The session ended with a general discussion on the future of characterization of complex traits. It was generally acknowledged that many tools – from genomics to high-throughput phenotyping – are now available that make it easier to map genes in both crop and wild species. The role of stable epigenetic changes in trait variation and its importance in wild and agricultural conditions remains unclear, and it is evident that more data as well as conceptual clarity in this area will be welcome. Finally, the growing information from plant systems biology provides opportunities to link genetic mapping with genetic networks, which could lead to greater understanding of the connections between genotypes and phenotypes.

## Domestication and Plant Improvement: Putting Science Into Practice in the Aid of the Human Condition

The third session of the meeting was devoted to the challenge of applying our understanding of plant genomic diversity and architecture to crop improvement. Maud Tenaillon (Centre National de la Recherche Scientifique, Ferme du Moulon, France) discussed recent work in her laboratory aimed at characterizing the genetic basis of flowering time in maize. She described the strategy used that consists of obtaining two maize populations from the same inbred line through divergent selection for flowering time. She showed that a significant response to selection in both directions was obtained. Her results point to few mutations with strong epistatic interactions as the genetic basis of the flowering time differences (Durand *et al.* 2015, 2012). Boulos Chalhoub (Unité de Recherche en Génomique Végétale, Institut National de la Recherche Agronomique, France) presented a progress report on the sequencing of the *Brassica napus* (oilseed rape) genome, an allopolyploid species resulting from a recent cross of *B. rapa* and *B. oleracea*. The *B. napus* genome reveals that there is substantial crosstalk between the two parental genomes, resulting in concerted homeologous exchanges, and shows that selection under domestication has resulted in a loss of undesirable glucosinolate genes and an expansion of oil biosynthesis genes (Chalhoub *et al.* 2014). Jenifer Hawkins (West Virginia University) described her laboratory's efforts to understand the impact of TEs in shaping the genome of *Sorghum*. She showed that TE abundance correlates with genome size, and that even a low level of TE activity generates small genome changes that can have a functional impact. Jordi Garcia-Mas (CRAG, Spain) presented his work on the characterization of QTL related to climacteric fruit ripening and the accumulation of sugars in melon fruits using genetic and genomic tools, such as the melon genome sequence and near isogenic lines (Argyris *et al.* 2015). Jim Giovannoni (Boyce Thompson Institute, Cornell University) explained the strategies followed by his laboratory toward elucidating molecular and genetic mechanisms underlying shelf-life and fruit quality. He gave an overview of the regulators of tomato ripening already discovered and presented new data relevant for continuing to improve tomato with respect to these two agronomically important characters (Klee and Giovannoni 2011; Gapper *et al.* 2013; Zhong *et al.* 2013). Rod Wing (Arizona Genetics Institute, University of Arizona) stressed the importance of the reservoir of natural variation in wild relatives of rice and presented an overview of efforts at the International Rice Research Institute to evaluate the phenotypes of some 3000 rice varieties. He also reported on progress of the iOMAP (international *Oryza* Map Alignment Project) consortium toward building reference genome assemblies for 11 wild *Oryza* species. Finally, Wing also presented the progress of his laboratory in dissecting the domestication of the African *O. glaberrima,* which took place independently of that of the Asian rice. He showed that in both species, the same shattering gene (*sh1*) was the target of human selection. The causal mutations are a SNP and a deletion for the Asian and the African species, respectively. Michael Purugganan (New York University) presented several population genomic analyses toward the characterization of several traits of agricultural importance, such as the analysis of 95 landraces of *O. glaberrima* to dissect the salt tolerance phenotype, and the analysis of the domestication of date palm through resequencing data of 62 varieties.

The session ended with a general discussion in which the notion of the pan genome and the dispensable (or optional) genome was discussed. The need for better genome sequences and standardized methods for genome annotation, in particular for TEs, was also emphasized during the discussion. Similarly, the need for good repositories of phenotypic data were also acknowledged. In summary, while during the last few years, whole genome data for crop species have become available, there is still a need for improvement of the sequences and their annotation, together with a proper description of the associated phenotypes to be able to bridge the gap between the genotype and the phenotype that will make possible a paradigm shift in crop improvement.

In total, the presentations at the conference revealed the extraordinary advances in our understanding of plant genome structure and the genesis of phenotypically relevant genetic variation. While connections to agronomically important phenotypes remain elusive in many cases, the meeting highlighted the promise of merging the often disparate disciplines of plant genomics and plant breeding, toward the goal of sustainably feeding a growing human population
